# Extramedullary Hematopoiesis in the Uterine Cervix Associated with Tissue Repair

**DOI:** 10.1155/2013/626130

**Published:** 2013-09-12

**Authors:** Suchanan Hanamornroongruang, Chanon Neungton, Malee Warnnissorn

**Affiliations:** ^1^Department of Pathology, Faculty of Medicine, Siriraj Hospital, Mahidol University, Bangkok 10700, Thailand; ^2^Department of Obstetrics and Gynecology, Faculty of Medicine, Siriraj Hospital, Mahidol University, Bangkok 10700, Thailand

## Abstract

Extramedullary hematopoiesis (EMH) is the presence of hematopoietic precursors outside the bone marrow. This condition is usually associated with hematologic disorders. Although EMH can be found in almost every site in the body, female genital tract involvement is rare. The authors report EMH in the uterine cervix from a 64-year-old patient following cervical biopsy due to abnormal cervical cytology. Neither neoplasm nor hematologic disorder was detected before the diagnosis and after 1 year of follow up. To the best of our knowledge, this is the first reported case of EMH involving the uterine cervix which showed an association with tissue repair.

## 1. Introduction

Extramedullary hematopoiesis (EMH) is the presence of hematopoietic precursors outside the medullary space of the bone marrow. Although this condition is physiologic in fetus, occurrence after birth usually associates with abnormal conditions, especially hematologic disorders. Common sites for pathologic EMH are the liver and spleen which are also normal sites for physiologic EMH in fetal life. Although EMH can be found in almost every site in the body, uterine involvement is rare. Thirty three cases of uterine EMH have been reported in the literature [1–8]. Only 4 cases showed cervical involvement [2, 5, 8], one was associated with myelofibrosis [5], and one was associated with chronic myeloid leukemia [8] whilst the remaining 2 cases showed no association with a significant hematologic disorder [2]. The authors reported a case of EMH involving the uterine cervix in association with tissue repair.

## 2. Case Presentation

A 64-year-old Thai female was referred to a gynecologist due to abnormal cervical cytology. Colposcopy and cervical biopsy were performed but no specific lesion was detected. Loop electrosurgical excision procedure (LEEP) for diagnosis was performed 2 weeks after biopsy.

**Figure 1:**
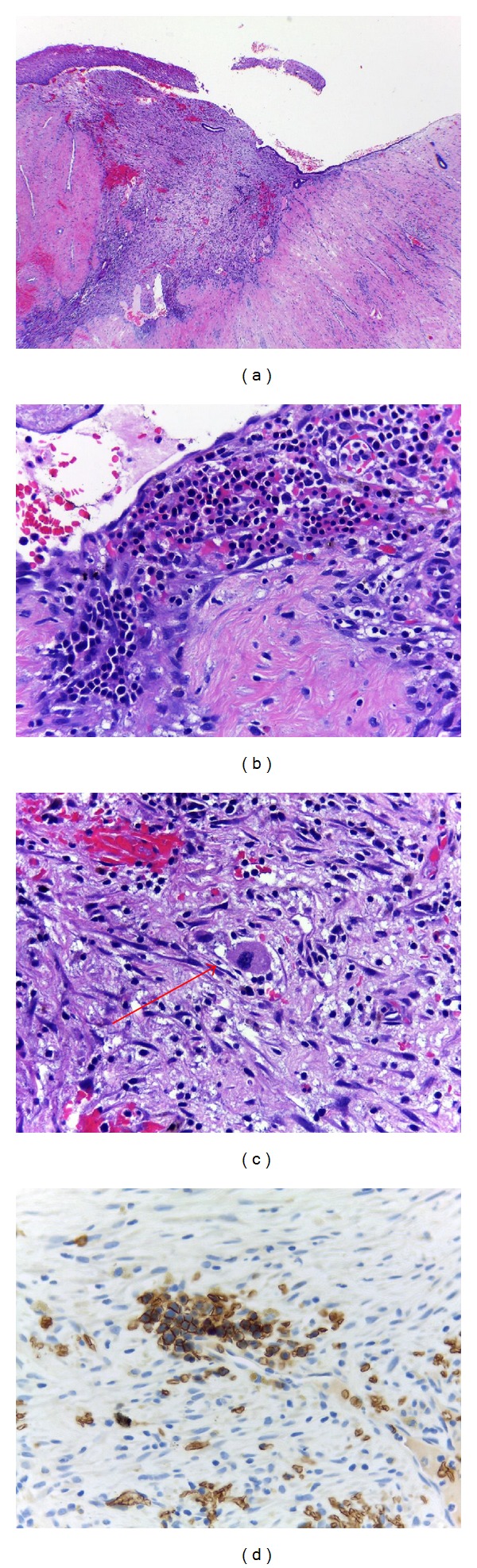
Microscopic appearance of the uterine cervix showed granulation tissue at squamocolumnar junction consistent with previous biopsy site (a). Clusters of erythroid precursors (b) and a megakaryocyte ((c), arrow) embedded in granulation tissue. The erythroid precursors were highlighted by glycophorin C (d).

Microscopic examination of cervical tissue revealed clusters of erythroids and few megakaryocytes embedded in granulation tissue consistent with previous biopsy site (Figures 1(a)–1(c)). The erythroid precursors were confirmed by glycophorin C (Figure 1(d)). 

Complete blood count of the patient was within normal limits (hemoglobin 13 g/dL, hematocrit 39.9%, red blood cell count 4.63 × 10^6^/uL, MCV 86 fl, MCH 28 pg, MCHC 33 g/dL, white blood cell count 6.4 × 10^3^/uL, and platelet count 264 × 10^3^/uL). Hemoglobin typing was normal. After detection of EMH, the patient remained well without evidence of neoplasm or hematologic disorder after a year of follow up.

## 3. Discussion

EMH usually arises secondary to other underlying disorders. Koch et al. showed that most of nonhepatosplenic EMH cases (92.6%) were associated with hematologic disorders; the most common condition (67%) was myelofibrosis with myeloid metaplasia [9]. In contrast, Gru et al. revealed that none of 20 uterine EMH cases had serious hematologic disorder other than chronic anemia [2]. There were other 13 reported cases of uterine EMH in the literature. Six were associated with hematologic disorders including 2 chronic myeloid leukemia [4, 8], and each of plasma cell myeloma [4], myeloproliferative disorder [4], myelofibrosis [5], and alpha thalassemia trait [4]. Three cases were associated with chronic endometritis [1]. Other associated conditions that have been reported were degenerated leiomyoma [3], adenosquamous carcinoma involving endometrium [1] metastatic breast carcinoma involving bone marrow [7], and retained product of conception [6] (Table 1). 

Four major theories explaining EMH include bone marrow failure, myelostimulation, abnormal systemic or local chemokine production, and tissue inflammation injury and repair [10]. This case report is in support of the latter theory. Similar finding by Hill and Swanson was observed in myocardial EMH, wherein 65% of myocardial specimens with EMH were associated with myocardial infarct of more than 72 hours [11]. According to this theory, circulating hematopoietic stem cells are thought to be attracted to site by cytokines and inflammatory mediators from the inflammation and repair process [10, 11]. Recently, Sun et al. successfully identified an adult uterine hemangioblast, a common precursor stem cell to hematopoietic and endothelial cell types in mouse [12]. If similar precursor stem cells exist in human, it may play a role in uterine EMH; further studies are needed.

EMH can be overlooked due to small sized hematopoietic cluster or obscuring inflammatory cells infiltrate and can be confused with other cells such as lymphocytes or histiocytes at low-power magnification. Recognition of this condition leads to proper clinical and laboratory investigations to rule out other serious underlying disorders, even though the majority of uterine EMH seem to be an incidental finding with no clinical significance. 

## Figures and Tables

**Table 1:** 

Reference no.	Author, year	No. cases	Location	Age (year)	Gynecologic condition	Hematologic condition
[1]	Sirgi et al., 1994	4	Endometrium	41–54	3 chronic endometritis 1 endometrial carcinoma with leiomyomas and adenomyosis	None

[2]	Gru et al., 2010	20	18 uterine fundus 2 cervix	27–75	55% disordered proliferative endometrium, 30% endometrial polyp, 45% leiomyoma, 15% adenomyosis, 15% chronic endometritis, 5% cervical endometriosis, 5% CIS of cervix, and 5% cervical high grade dysplasia	60% anemia

[3]	Schmid et al., 1990	1	Uterine mass	66	Degenerating leiomyoma	None

[4]	Creagh et al., 1995	4	3 endometrium	43–68	2 menorrhagia (proliferative endometrium)	Myeloproliferative disorder
						Chronic myeloid leukemia
					1 endometrial stromal sarcoma	Alpha-thalassemia trait
			1 endomyometrium		1 incidental finding from autopsy	Plasma cell myeloma

[5]	Pandey et al., 1999	1	Cervix	60	CIN II	Myelofibrosis

[6]	Valeri et al., 2002	1	Endometrium	23	Retained products of conception	Mild anemia

[7]	Varras et al., 2002	1	Uterine isthmus	40	Right ovarian cyst	Bone marrow infiltration by breast carcinoma

[8]	Palatnik et al., 2012	1	Cervix, endometrium ovaries	43	Bilateral tubo-ovarian masses	Chronic myeloid leukemia

	Present case	1	Cervix	64	LEEP following a negative cervical biopsy Prior abnormal cervical cytology	None
